# Excito-Secretory System

**Published:** 1858

**Authors:** 


### Excito-Secretory System.

Marshall Hall, sometime since, avowed the existence of such a system as above, whose physiological office was supposed to be similar to that of the excito-motory. Since then, Dr. Campbell, of Georgia, has appeared in a pamphlet, establishing quite clearly the priority in the discovery, or rather the first to suggest the theory. Since his pamphlet, again, Prof. Allen of Michigan has appeared with an article in the Medical Independent and has as fully established the fact that he taught similar doctrines, if not the identical theory, as maintained by the first two. The article from his pen is able and interesting, and we will be pleased to publish it in a future number, for the benefit of our readers. There could have been no plagiarism in this matter, but seems to have been a coincidence purely. If the claimants to originality were not already numerous enough, another might be added to the list, for the occupant of the Chair of Physiology and Institutes in this Institution made similar suggestions three years ago, and has repeated them several times since, as the members of the several classes can testify. It was avowed as an original thought, in connection with his own theory of the capillary circulation, not, however, as having any direct relation, but simply that both were original, and simply suggestions.

